# STIM1 Phosphorylation at Y361 Recruits Orai1 to STIM1 Puncta and Induces Ca^2+^ Entry

**DOI:** 10.1038/srep42758

**Published:** 2017-02-20

**Authors:** Pascal Yazbeck, Mohammad Tauseef, Kevin Kruse, Md-Ruhul Amin, Rayees Sheikh, Stefan Feske, Yulia Komarova, Dolly Mehta

**Affiliations:** 1 Department of Pharmacology and Center for Lung and Vascular Biology, University of Illinois College of Medicine, Chicago, IL 60612, USA; 2 Department of Pharmaceutical Sciences, College of Pharmacy, Chicago State University, Chicago, IL 60628, USA; 3 Department of Pathology, New York University School of Medicine, New York, NY 10016, USA

## Abstract

Store-operated Ca^2+^ entry (SOCE) mediates the increase in intracellular calcium (Ca^2+^) in endothelial cells (ECs) that regulates several EC functions including tissue-fluid homeostasis. Stromal-interaction molecule 1 (STIM1), upon sensing the depletion of (Ca^2+^) from the endoplasmic reticulum (ER) store, organizes as puncta that trigger store-operated Ca^2+^ entry (SOCE) *via* plasmalemmal Ca^2+^-selective Orai1 channels. While the STIM1 and Orai1 binding interfaces have been mapped, signaling mechanisms activating STIM1 recruitment of Orai1 and STIM1-Orai1 interaction remains enigmatic. Here, we show that ER Ca^2+^-store depletion rapidly induces STIM1 phosphorylation at Y361 via proline-rich kinase 2 (Pyk2) in ECs. Surprisingly, the phospho-defective STIM1-Y361F mutant formed puncta but failed to recruit Orai1, thereby preventing. SOCE Furthermore, studies in mouse lungs, expression of phosphodefective STIM1-Y361F mutant in ECs prevented the increase in vascular permeability induced by the thrombin receptor, protease activated receptor 1 (PAR1). Hence, Pyk2-dependent phosphorylation of STIM1 at Y361 is a critical phospho-switch enabling recruitment of Orai1 into STIM1 puncta leading to SOCE. Therefore, Y361 in STIM1 represents a novel target for limiting SOCE-associated vascular leak.

Endothelial barrier function is vital in the regulation of tissue-fluid homeostasis, angiogenesis, and inflammation^1^,^2^. Loss of endothelial barrier function following burn, trauma, or sepsis leads to acute lung injury (ALI), a life threatening condition due to respiratory failure^3^,^4^. A rise in intracellular free calcium concentration ([Ca^2+^]i) caused by edemagenic agonists such as thrombin induces actin-myosin contraction in endothelial cells (ECs) leaving wide gaps between adjacent ECs^5^,^6^. These gaps, if not sealed, lead to elevated vascular permeability resulting in debilitating pulmonary edema. It is well-established that store-operated calcium entry (SOCE), activated upon depletion of Ca^2+^ from ER stores, plays a critical role in disrupting endothelial barrier function^7^,^8^.

STIM1 has been recently identified as a fundamental sensor of the Ca^2+^ concentration inside the ER lumen and thereby regulates SOCE in various cell types including ECs^9^,^10^,^11^. It is a multi-domain protein containing a single transmembrane α-helix^12^. The N-terminus is located inside the ER lumen and consists of a Ca^2+^ binding EF hand and a sterile α-motif (or SAM domain)^13^,^14^,^15^. The transmembrane domain of STIM1 is flanked by an ezrin-radixin-moesin (ERM) motif known to interact with F-actin filaments^16^. STIM1’s C-terminus is composed of three coiled-coil (CC) domains, a COOH-terminal inhibitory domain (CTID), serine-proline rich (S/P) and threonine-arginine-isoleucine-proline (TRIP) regions and a lysine rich region (K-region)^14^. Mutational and molecular modeling analysis identified a stretch consisting of ~100 amino acids, from 340 to 440 aa, spanning the CC2 and CC3 domains, referred to as STIM1–Orai1 activating region (SOAR) (also known as CRAC activating domain, CAD or CCb9), as an essential domain for STIM1 oligomerization and activation of SOCE via Orai1^17^,^18^,^19^,^20^.

Orai1 is the predominant pore-forming subunit in highly calcium-selective channels known as Ca^2+^-release activated Ca^2+^ channels (CRAC) in various cell types including ECs^21^. Thus, the current model of SOCE activation by STIM1 indicates that upon store depletion, STIM1 forms puncta. Puncta are defined as discrete structures formed near the endoplasmic reticulum-plasmamembrane (ER-PM) junctions through aggregation of STIM1 molecules following Ca^2+^ store depletion in the ER^22^. Hence, upon store depletion, STIM1 undergoes conformational changes within its EF-SAM domains allowing long-range regulation within the CC1 domain that extends and organizes the SOAR domain to form discrete puncta^23^. These puncta are then recruited towards the plasma membrane where STIM1 interacts with Orai1 to trigger SOCE^24^,^25^. The fundamental question whether the interaction between the STIM1 and Orai1 is regulated by intracellular signaling remains unanswered. STIM1 was discovered as a phosphoprotein^26^. Studies so far have focused on the role of phosphorylation of serine residues located within the S/P stretch of STIM1 in dissociating STIM1 from microtubule end-binding protein as a mechanism of STIM1 modulation of Ca^2+^ entry and cell proliferation^26^,^27^,^28^,^29^,^30^,^31^,^32^,^33^.

The STIM1 dimer, which organizes to form puncta, has a predicted V-shaped tertiary structure in which the Y361 residue contributed by each monomer are closely apposed (~1.5 A^0^ apart)^25^,^34^,^35^,^36^. Whether STIM1 Y361 is regulated post-translationally by intracellular signaling and thereby modulates the interaction between the STIM1 and Orai1 remains unanswered. Here, we identified a key role of Pyk2-dependent phosphorylation of STIM1 at Y361 in STIM1 gating of Orai1 and thereby SOCE. We show for the first time that while tyrosine phosphorylation of STIM1 at Y361 is not required for STIM1 puncta formation, it is essential for recruiting Orai1 into STIM1 puncta to trigger SOCE. Furthermore, we established the physiological role of STIM1 phosphorylation in regulating endothelial permeability *in vivo* by demonstrating that selective transduction of the phospho-defective STIM1-Y361F mutant into the pulmonary endothelium of WT mice blocked the increase in endothelial permeability induced by activation of the thrombin receptor, protease activated receptor 1 (PAR1).

## Results

### Phosphorylation of STIM1 at Y361 is required to trigger SOCE

**Figure 1:**
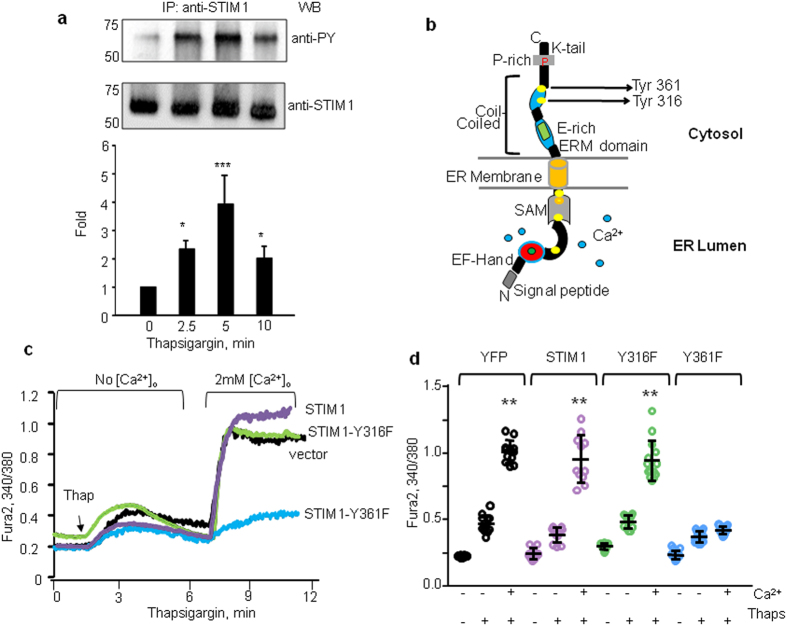
Thapsigargin induces phosphorylation of STIM1 at Y361 residue to facilitate SOCE. **(a**) STIM1 was immunoprecipitated (IP) with anti-STIM1 antibodies from lysates of HPAE cell treated with 2 μM thapsigargin for indicated time points. Resulting precipitates were probed with the phosphotyrosine antibodies. A representative immunoblot (top) and densitometry of bar graph of mean ± SD (bottom) demonstrate transient changes in STIM1 phosphorylation on tyrosine residues at various times after thapsigargin stimulation. Fold change was expressed as a ratio between phosphotyrosine and total STIM1 density normalized to 0 times. Data are from 3 independent experiments. *p < 0.05, and ***p < 0.001 indicates significant increase as compared to 0 time points. **(b**) Schematic representation of STIM1 domains and putative tyrosine phosphorylation sites. Note, Y316 and 361 are located in Cα2 and SOAR domains, respectively. (**c**,**d**) Phosphorylation of STIM1-Y361 residue is required for inducing Ca^2+^ entry. HPAE cells were transfected with vector (YFP), WT-STIM1, Y316F-STIM1 or Y361F-STIM1 mutants. Cells were stimulated with thapsigargin in Ca^2+^ -free medium followed by addition of 2 mM Ca^2+^ at ~300 s. (**c**) Representative traces are shown.. (**d)** Individual data points (from 5–10 cells) and mean ± SD are plotted from 3 independent experiments. **p < 0.01 compared to unstimulated cells or cells stimulated with thapsigargin in Ca^2+^ -free medium.

STIM1 contains a number of tyrosine residues^37^ two of which lie within SOAR domain but the role of these potential phosphorylation sites in regulating SOCE remains unclear. Here, we addressed the question whether thapsigargin, an indirect activator of SOCE through ER store depletion, induces tyrosine phosphorylation of STIM1. Human pulmonary aortic endothelial (HPAE) cells were stimulated with thapsigargin at the indicated time points (Fig. 1a) and STIM1 was immunoprecipitated from the cell lysates using an anti-STIM1 antibody. Immunocomplexes were then probed with anti-phosphotyrosine (anti-PY) antibodies to detect tyrosine phosphorylation. We observed that STIM1 was minimally phosphorylated on tyrosine residues in naïve HPAE monolayers whereas thapsigargin promptly stimulated the phosphorylation of STIM1 within 2.5 min. The phosphorylation levels peaked (4-fold increase) at 5 min (Fig. 1a). Hence, our data demonstrate that STIM1 undergoes phosphorylation on tyrosine residues, which coincides with Ca^2+^ release from ER stores.

The phosphopeptide analysis of STIM1 (http://www.cbs.dtu.dk/services/NetPhos-2.0/) predicted phosphorylation of six tyrosine residues (Fig. 1b, yellow circles). Intriguingly, Y361 and another residue, Y316, located within the second α-helix (also known as Cα2) of the coil-coil domains were predicted to be two key phospho residues (Fig. 1b). Thus, to investigate whether phosphorylation of either Y316 or Y361 is required for SOCE, we introduced a single point mutation at Y316 or Y361 by substitution of phenylalanine (316Y** → **316F; 361Y** → **361F) to generate phospho-defective STIM1 mutants and tagged them with YFP. We first stimulated ECs with thapsigargin in Ca^2+^ free media to induce Ca^2+^ release from ER (first peak) followed by repletion of 2 mM extracellular Ca^2+^ to activate SOCE (second peak). Overexpression of STIM1-WT or the phospho-defective STIM1-Y316F mutant had no effect on Ca^2+^ release or SOCE as compared to cells expressing control vector (Fig. 1c,d, and Supplementary Figure 1a). Intriguingly, overexpression of the phospho-defective STIM1-Y361F mutant did not alter Ca^2+^ release but suppressed Ca^2+^ entry by ~85% (Fig. 1c,d, and Supplementary Figure 1a) indicating that tyrosine phosphorylation of STIM1 at Y361 plays a key role in regulating SOCE. To confirm that Y361 in STIM1 is a primary phosphorylation site, we overexpressed STIM1-WT and the STIM1-Y361F mutant in human embryonic kidney (HEK) cells and determined whether mutation at Y361 alters STIM1 phosphorylation in response to thapsigargin (Supplementary Figure 1b). Again, we detected the phosphorylation of WT-STIM1 but modest phosphorylation of STIM1-Y316F mutant with anti-PY antibodies indicating that Y361 is the main tyrosine phosphorylation site in STIM1 (Supplementary Figure 1b). We concluded, therefore, that STIM1 undergoes transient phosphorylation on its Y361 residue, which in turn, promotes SOCE.

### Pyk2 phosphorylates STIM1

**Figure 2:**
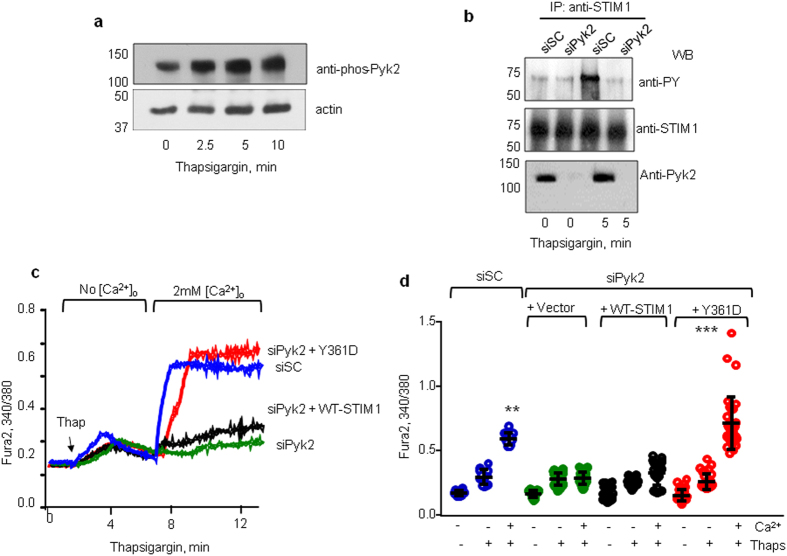
Pyk2 phosphorylates STIM1. (**a)** Western blot analysis of a time-course of Pyk2 phosphorylation using anti-Y-402-phospho-Pyk2 in HPAE cells treated with thapsigargin at indicated time points. Note, Pyk2 undergoes phosphorylation after treatment with thapsigargin. A representative blot is shown from experiments that were repeated at least three times. **(b)** Depletion of Pyk2 blocks STIM1 tyrosine phosphorylation. Western blot analysis of STIM1 phosphorylation (as described in Fig. 1a) in HPAE cells depleted of Pyk2 and treated with thapsigargin at indicated time points. Western blot with anti-Pyk2 was used to determine efficiency of Pyk2 depletion. siSC, scramble siRNA; siPyk2, Pyk2-targeting siRNA. A representative blot is shown from experiments that were repeated at least three times. (**c**,**d)** Overexpression of phospho-mimetic Y361D-STIM1 mutant but not WT-STIM1 or phosho-defective Y361F-STIM1 mutant rescues SOCE in Pyk2-depleted cells. Effect of Pyk2 depletion on SOCE was assessed as in Fig. 1. Representative traces are shown (**c**). (**d**) Summary results. Individual data points (from 5–10 cells) and mean ± SD are plotted from 3 independent experiments. **p < 0.01, ***p < 0.001 compared to unstimulated cells or cells stimulated with thapsigargin in Ca^2+^-free medium.

Next, we sought to determine the kinase responsible for STIM1 tyrosine phosphorylation. Proline-rich tyrosine kinase 2 (Pyk2) is regulated by intracellular Ca^2+^ in several cell types including ECs^38^,^39^,^40^. We, therefore, assessed if thapsigargin induces Pyk2 activity, which in turn, regulates tyrosine phosphorylation of STIM1. Using a Pyk2 phospho-Y402-specific antibody^40^,^41^, we demonstrated that thapsigargin rapidly activated Pyk2 in ECs (Fig. 2a). However, thapsigargin did not activate other tyrosine kinases such as p60Src (data not shown). To establish the role of Pyk2 in inducing STIM1 phosphorylation and thereby SOCE, we depleted Pyk2 using siRNA. Additionally, we assessed if rescuing STIM1 phosphorylation at Y361 in Pyk2-depleted ECs rescues SOCE. We found that depletion of Pyk2 markedly suppressed STIM1 phosphorylation in ECs in response to thapsigargin (Fig. 2b).

Depletion of Pyk2 also reduced SOCE (Fig. 2c,d) mimicking findings in cells transducing the STIM1-Y361F mutant. Furthermore, SOCE was restored in Pyk2 depleted cells by rescuing STIM1 phosphorylation through overexpression of the phospho-mimetic STIM1-Y361D mutant (Fig. 2c,d). These data identify Pyk2 as the kinase phosphorylating STIM1 on its Y361 residue, activating thereby SOCE.

### STIM1 phosphorylation of Y361 is required for STIM1-Orai1 interaction and recruitment of Orai1 to STIM1 puncta

**Figure 3:**
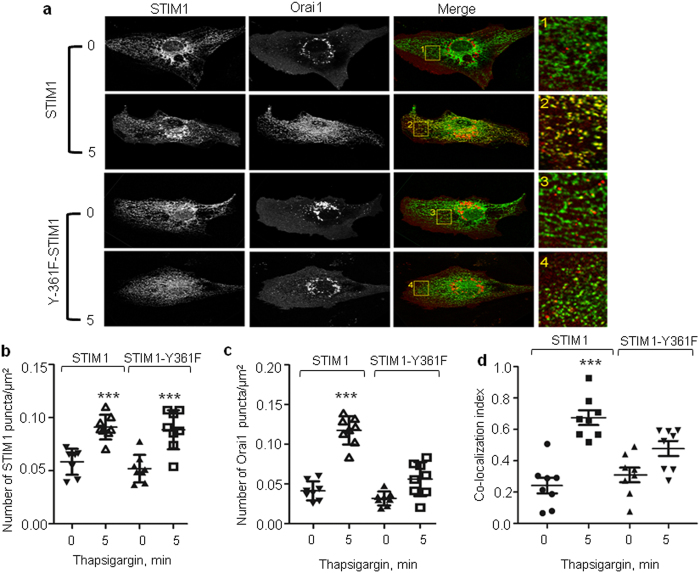
Tyrosine phosphorylation of STIM1 at Y361 residue mediates recruitment of Orai1 to STIM1 puncta. (**a**,**d)** HPAE cells co-expressing either WT-YFP-STIM1 and mCherry-Orai1 or Y361F-YFP-STIM1 and mCherry-Orai1 were stimulated with 2 μM thapsigargin for the indicated time points, fixed and visualized with confocal LSM 880 microscope. Images showing STIM1 puncta and recruitment of Orai1 to STIM1 puncta **(a)**, number of STIM1 **(b)** and Orai1 clusters **(c)**, colocalization index for Orai1 **(d).** Individual data points (from 5–10 cells) and mean ± SD are plotted. ***p < 0.001 compared to unstimulated cells **(b**–**d)** or thapsigargin stimulated STIM1-Y361F mutant expressing cells **(c,d)**.

Studies show that upon store depletion, STIM1 appears in discrete puncta within the ER membrane that recruit Orai1 following translocation to ER-PM junctions^42^. Because Y361 residues are critically positioned within the STIM1 dimer^25^,^34^,^36^, we asked if phosphorylation of STIM1 might be required to induce conformational changes within STIM1 to trigger STIM1 multi-dimerization or interaction with Orai1. To test this possibility, we analyzed the intracellular distribution of both WT-STIM1 and the phospho-defective Y361F-STIM1 mutant with respect to WT-Orai1 in ECs following thapsigargin stimulation. Interestingly, thapsigargin increased puncta formation both in cells expressing WT-STIM1 and those expressing the STIM1-Y361F mutant, suggesting that phosphorylation of the Y361 residue is not required for STIM1 multi-dimerization (Fig. 3a,b). In contrast, increase in the number of Orai1 puncta by thapsigargin was significantly reduced in cells expressing the STIM1-Y361F mutant (Fig. 3a,c). Also, Orai1 failed to co-localize with the STIM1-Y361F mutant (Fig. 3a,d) suggesting that STIM1 phosphorylation dynamically regulates recruitment of Orai1 to the STIM1 puncta where they can interact.

**Figure 4:**
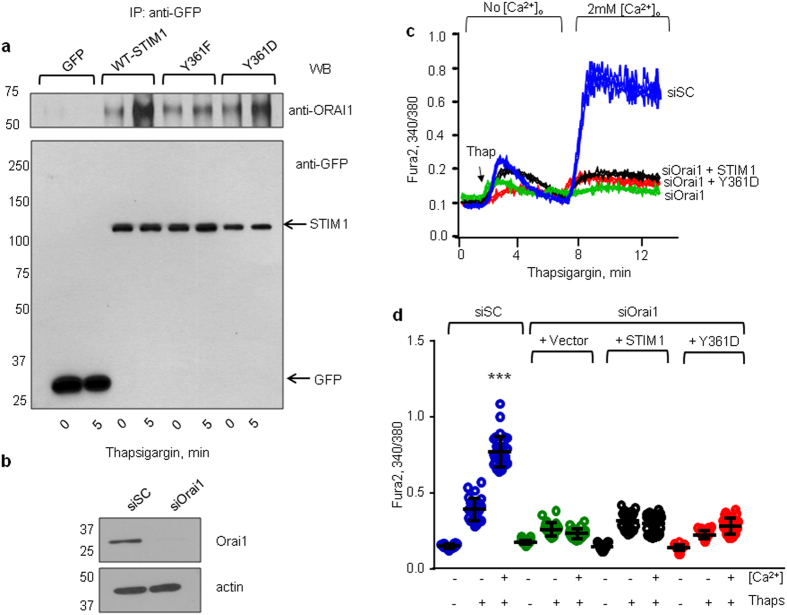
Tyrosine phosphorylation of STIM1 at Y361 residue mediates STIM1-Orai1 interaction and activation of Orai1. **(a)** HEK cells co-expressing YFP, WT-YFP-STIM1, or YFP-Y361F-STIM1, YFP-Y361D-STIM1 mutant together with mCherry-Orai1 were stimulated with thapsigargin for 5 min or left unstimulated. Exogenous STIM1 was immunoprecipitated with anti-GFP antibodies followed by immunoblotting using both anti-Orai1 and anti-GFP antibodies. A representative blot is shown from experiments that were repeated multiple times. (**b)** Western blot analysis of Orai1 expression in HPAE cells depleted of Orai1. A representative blot is shown from experiments that were repeated multiple times. siSC, scramble siRNA; siOrai1, Orai1-targeting siRNA. **(c,d)** Overexpression of Y361D-STIM1 mutant failed to rescue SOCE in Orai1 depleted ECs. Effect of Orai1 depletion on SOCE was assessed as in Fig. 1c,d; (**c**) Representative traces are shown; (**d**) Summary results. Individual data points (from 10–15 cells) and mean ± SD are plotted from 3 independent experiments. ***p < 0.001 compared to unstimulated cells or thapsigargin stimulated cells as indicated (**c**,**d**).

To further corroborate the role of STIM1 phosphorylation on Y361 in inducing interaction with Orai1, we performed immunoprecipitation of Orai1 in HEK cells expressing either WT, phospho-defective (STIM1-Y361F), or phospho-mimetic (STIM1-Y361D) mutants. We found that thapsigargin induced the interaction of Orai1 with WT-STIM1 (Fig. 4a). However, thapsigargin failed to induce an interaction between Orai1 and the STIM1-Y361F mutant (Fig. 4a). Cells transducing the Y361D-STIM1 mutant behaved similar to WT-STIM1 expressing cells, i.e. we did not observe any greater interaction of this mutant with Orai1 basally (Fig. 4a). Next, we depleted Orai1 in ECs, which reduced Orai1 expression by more than 90% (Fig. 4b). We then expressed WT-STIM1 or the phospho-mimetic STIM1 mutant (Y361D-STIM1 mutant) into Orai1 depleted cells and imaged YFP-tagged cells to establish the role of STIM1 activation of Orai1 as a requisite for SOCE. We found that both WT-STIM1 and the Y361D-STIM1 mutant failed to rescue SOCE in Orai1 depleted ECs (Fig. 4c) supporting the contention that phosphorylation of STIM1 at Y361 induces STIM1-Orai1 interaction and Orai1 gating.

### STIM1 phosphorylation at Y361 residue is required for increasing vascular permeability

**Figure 5:**
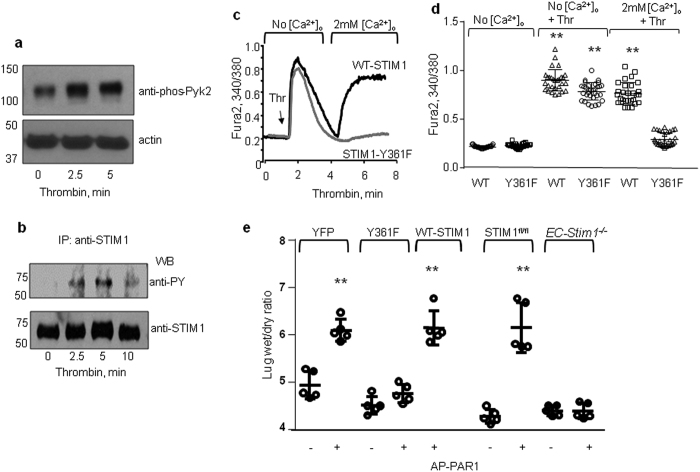
STIM1 phosphorylation at Y361 residue contributes to increased lung vascular permeability. (**a)** Western blot analysis of a time-course of Pyk2 phosphorylation using anti-Y-402-phospho-Pyk2 in HPAE cells treated with thrombin as determined in Fig. 1. Image shown are cropped. Full immunoblot is uploaded in Supplementary Figure S3. A representative blot is shown from experiments that were repeated multiple times (**b)** Thrombin induces tyrosine phosphorylation of STIM1. Lysates were IP with anti-STIM1 antibody. Resulting precipitates were probed with the phosphotyrosine antibodies. A representative blot is shown from experiments that were repeated multiple times. (**c**,**d)** Phosphorylation of STIM1 on Y361 is required for α-thrombin-induced SOCE. A representative trace **(c).** (**d**) Individual data points (from 10–15 cells) and mean ± SD are plotted from 3 independent experiments. **p < 0.01 compared to unstimulated cells or thrombin stimulated STIM1-Y361F mutant expressing cells. (**e**) Liposomes containing either YFP, WT-STIM1 or Y361F-STIM1 were injected into WT mouse ice through retroorbital sinus 40 h prior to experiments. In parallel experiment, STIM^fl/fl^ and *EC-Stim1*^*−/−*^ were used. To induce vascular permeability, PAR1 agonist peptide was given i.v. 30 min prior to harvesting lungs for measurement of lung wet/dry ratio. Individual data points and mean ± SD are plotted from 5 mice. Experiments were repeated two times. **p < 0.01 compared to mice receiving control peptide or PAR1 activated mice expressing STIM1-Y361F mutant.

We next addressed the physiological significance of STIM1 phosphorylation on its Y361 residue in regulating increased endothelial permeability. We first determined whether inhibition of STIM1 phosphorylation altered SOCE secondary to activation of the G-protein coupled receptor PAR1. Similar to thapsigargin, activation of PAR1 with thrombin also increased Pyk2 and STIM1 phosphorylation in a time dependent manner (Fig. 5a,b). Thrombin induced a similar Ca^2+^ release in cells transducing WT-STIM1 or control vector (data not shown) or phospho-defective STIM1-Y361F mutant (Fig. 5c,d, first peak). However, thrombin induced SOCE in cells transducing WT-STIM1 but not in cells expressing the phosphor-defective STIM1-Y361F mutant (Fig. 5c,d, second peak). To address the role of STIM1 phosphorylation in regulating endothelial permeability *in vivo*, we used liposome-based gene delivery. This approach effectively induces the expression of gene of interest in endothelium which remains functional over next 72h^43^,^44^,^45^. Thus, we prepared cationic liposomes containing either control vector driven by the VE-cadherin promoter for YFP (empty vector control), WT, or the phosphor-defective STIM1-Y361F mutant. These liposomes were injected intravenously into mice to determine the role of STIM1 phosphorylation in increasing pulmonary vascular permeability. Expression of YFP, WT-STIM1 and the STIM1-Y361F mutant in lung microvasculature was confirmed with western blot analysis (Supplementary Figure 2a). As expected, a selective PAR1 agonist peptide increased lung vascular permeability in mice transducing vector alone or WT-STIM1 (Fig. 5e). However, the PAR1-agonist peptide failed to increase vascular permeability in mice transducing the STIM1-Y361F mutant (Fig. 5e). In other studies, we assessed vascular permeability in response to PAR1 activation in mice lacking STIM1 only in ECs (Supplementary Figure 2b). We found that PAR1 peptide increased vascular permeability in STIM1-floxed (control) mice to a level similar to WT mice receiving liposomes containing YFP or WT-STIM1 vectors (Fig. 5e). However, activation of PAR1 failed to increase vascular permeability in mice lacking STIM1 in ECs (*EC-Stim1*^*−/−*^ *mice;* Fig. 5e). The block of vascular permeability increase in mice transducing the phospho-defective STIM1-Y361F mutant was similar to that of mice lacking STIM1 in ECs (*EC-Stim1*^*−/−*^ *mice*). These findings thereby demonstrate a pivotal role of STIM1 phosphorylation on Y361 in mediating the lung vascular permeability response.

## Discussion

In this study, we have uncovered a novel regulatory mechanism that primes STIM1 to interact with the Ca^2+^-selective Orai1 channel inducing SOCE. In this context, Pyk2-dependent phosphorylation of the Y361 residue within STIM1 induces SOCE. Our data showed that phosphorylation of the Y361 residue does not interfere with STIM1 puncta formation but controls recruitment of Orai1 to STIM1 puncta enabling STIM1-Orai1 interaction and Ca^2+^ entry through Orai1 channels. Furthermore, we established the physiological role of STIM1 tyrosine phosphorylation in regulating endothelial permeability *in vivo* by demonstrating that transduction of the phospho-defective STIM1-Y361F mutant in pulmonary endothelium of WT mice blocked the increase in endothelial permeability associated with activation of PAR1 signaling.

Both STIM1 and Orai1 are integral components of SOCE in ECs^21^,^46^. STIM1 binds Ca^2+^ in the ER lumen through its EF hand motif under basal conditions^36^,^47^. Several inflammatory mediators such as thrombin, histamine, and growth factors initiate ER-Ca^2+^ depletion, which is followed by Ca^2+^ entry through SOCE^48^. Ca^2+^ entry is a multistep process, which requires sensing of the Ca^2+^ concentration within the ER, establishment of ER- plasmalemmal (PM) junctions, and activation of plasmalemmal Ca^2+^ channels. STIM1 plays a critical role in each of these steps^49^. It functions as a sensor for Ca^2+^ concentration inside the ER lumen. Following agonist-induced depletion of the ER-Ca^2+^ store, STIM1 oligomerizes to form numerous puncta within the ER membrane^42^. These puncta then translocate to the ER-PM junctions where STIM1 interacts with plasmalemmal Ca^2+^ channels such as Orai1 channels to activate SOCE^50^.

In the present study, we showed that Pyk2-dependent tyrosine phosphorylation of STIM1 at Y361 within SOAR domain of STIM1 is an integral step in activating Ca^2+^ entry through Orai1 channels. We showed that Pyk2 but not p60Src is rapidly activated following stimulation of ECs with the SERCA inhibitor thapsigargin or thrombin. Depletion of Pyk2 inhibited both STIM1 phosphorylation and Ca^2+^ entry in ECs, demonstrating that Pyk2 mediates phosphorylation of STIM1. We showed that, while the expression of the phospho-defective STIM1-Y361F mutant markedly suppressed Ca^2+^ entry in ECs in response to either thapsigargin or thrombin, expression of the phospho-mimetic STIM1-Y361D mutant was sufficient to restore SOCE in Pyk2-depleted ECs, thus indicating that activation of Pyk2 through Ca^2+^ mobilization from ER stores provides a positive feedback mechanism for Ca^2+^ entry by phosphorylating STIM1.

Our findings also showed that the phospho-defective STIM1-Y361F mutant exhibited a dominant negative effect through inactivation of endogenous STIM1. We speculate that the ectopically-expressed STIM1-Y361F mutant blocks SOCE by forming dysfunctional STIM1 oligomers with endogenous STIM1 protein upon ER-Ca^2+^ depletion. This impairs recruitment of Orai1 into STIM1 puncta, limiting formation of functional Orai1 channels. Our findings also showed that recruitment of Orai1 into STIM1 puncta is a critical step in inducing Ca^2+^ entry via the SOCE, since expression of the STIM1-Y361F mutant or depletion of Orai1 in ECs inhibited SOCE to a similar degree.

Crystal structure analysis revealed that the STIM1 dimer forms a V-shaped structure in which the Y361 residue from each monomer meet at the angle of the “V” shaped structure^34^. We therefore surmised that phosphorylation of Y361 might be required for generation of repulsive forces within the “V” shaped structure, inducing both STIM1 puncta formation and interaction with Orai1. To our surprise, phosphorylation of Y361 was not required for STIM1 dimerization since a loss-of-function of STIM1 (Y361F-STIM1 mutant) did not alter the number of STIM1 puncta as compared to WT-STIM1 upon thapsigargin stimulation. However, phosphorylation of Y361 was found to be required for recruiting and ’trapping’ Orai1 by STIM1 puncta at the plasma membrane. These findings support a recently described ‘trafficking trap’ hypothesis in which cortical STIM1 puncta govern Orai1 recycling at the plasma membrane^51^. Interestingly, our data also showed that a gain-of-function STIM1 mutation *per se* did not increase STIM1’s interaction with Orai1 basally indicating that phosphorylation of STIM1 alone is not sufficient to promote STIM1 and Orai1 interaction at the ER membrane and that consequently, STIM1 reorganization into puncta is a prerequisite for this interaction. We, therefore, conclude from these findings that recruitment of Orai1 into STIM1 puncta is an independent step requiring STIM1 phosphorylation by Pyk2.

How phosphorylation of Y361 regulates STIM1’s interaction with Orai1 remains to be parsed out but some speculation may be offered. Studies have identified SOAR as the minimal domain required for both STIM1 puncta formation and activation of Orai1 channels^25^,^34^,^36^. However, full-length STIM1 is inactive as steric hindrance from its CC1 domain prevents unfolding of the STIM1-SOAR domain^52^. Structural studies showed that the SOAR domain interacts with Orai1’s C-terminus through the STIM1-Orai1 association pocket (SOAP)^42^,^53^. This pocket is formed through coupling of several positively charged lysine residues from the STIM1-CC2 domain with negatively charged (D284, D287, D291) residues on Orai1^54^. It is possible, therefore, that STIM1 phosphorylation on Y361 may expose positively charged lysine residues in STIM1 enabling recruitment and interaction of Orai1 with STIM1.

Activation of SOCE by STIM1 plays a key role in regulating endothelial permeability^55^,^56^,^57^. We showed that PAR1 activation caused an increase in endothelial permeability in the lungs of WT mice expressing either the YFP empty vector or WT-STIM1. However, expression of the phospho-defective mutant (Y361F-STIM1 mutant) prevented the increase in vascular permeability by PAR1 much like the findings in mice lacking STIM1 in ECs. These data establish a critical role of the STIM1 phospho-switch in disrupting endothelial barrier function. Together, our data suggest a model in which phosphorylation of STIM1 on its Y361 residue represents an independent and obligatory step linking STIM1 puncta to gating of Orai1 channels and thereby Ca^2+^ entry and increased endothelial barrier permeability. This step is required for recruitment of Orai1 into STIM1 puncta and STIM1-Orai1 interaction. Our observations provide the first direct evidence for tyrosine phosphorylation-dependent activation of SOAR that mediates recruitment of Orai1 to STIM1 puncta. This phospho-switch is essential for activation of Ca^2+^ entry through Orai1 and disruption of endothelial barrier function in lungs, which leads to several pathologic disorders including lung injury. In this sense, STIM1 phsophorylation at Y361 represents a novel target for preventing diseases associated with leaky blood vessels.

## Methods

### Materials

FUGENE transfection reagent was purchased from Promega (Madison, WI, USA). Primary antibodies against phospho-tyrosine (PY20, PY99, and PY350), Orai1, Stim1, GFP as well as A/G agarose beads were purchased from Santa Cruz Biotechnology (Santa Cruz, CA, USA). Phospho-Pyk2 and pan Pyk2 antibodies were purchased from Cell Signaling Technologies (Beverly, MA, USA). Thapsigargin was purchased from EMD Millipore (MA, USA). Paraformaldehyde was purchased from Fisher Scientific (Hampton, NH, USA). Fura 2-AM was purchased from Life Technologies (Carlsbad, CA, USA). Scrambled and Pyk2 siRNA were purchased from Ambion (Foster City, CA). All details are described in Supplemental Experimental Procedures.

### Cell culture

Human pulmonary arterial endothelial cells (HPAECs) and EBM-2 medium were purchased from (Lonza, Allendale, NJ, USA). HPAECs were cultured in complete EBM-2 medium in a T-75 flask coated with 0.1% gelatin and supplemented with 10% fetal bovine serum (FBS) and maintained at 37 °C in a humidified atmosphere of 5% CO_2_ and 95% air until they formed a confluent monolayer. Cells were detached using 0.05% Trypsin containing 0.02% EDTA and plated on 100 mm dishes. HEK cells were plated on 100 mm dishes and cultured using DMEM medium supplemented with 10% fetal bovine serum (FBS) till confluent.

### Transfection

ECs from the primary flask were detached using 0.05% Trypsin containing 0.02% EDTA. HPAEC were transfected with cDNA using FUGENE HD Transfection Reagent (Promega, Madison, WI) or Amaxa electroporation (Lonza) as described^6^. In experiments requiring siRNA, cells were transfected with scrambled or indicated siRNA using Santa Cruz transfection reagent or Amaxa electroporation. Confluent monolayers were incubated in serum-free MCDB-131 medium for 1–2 h before stimulation with thapsigargin or thrombin. HPAEC were used between passages 6 and 8. HEK cells were transfected upon reaching 80% confluency with Fugene for 48 h following the manufacturer’s protocol. Media was changed after 24 h transfection.

### Immunoprecipitation

Cells were lysed in radioimmunoprecipitation buffer (50 mM Tris, pH 7.4, 1% deoxycholic acid, 150 mM NaCl, 0.25 mM EDTA, pH 8.0, 0.5% Nonidet P-40, 0.1% SDS, 1 mM NaF, 1 mM sodium orthovanadate,1 mM phenylmethylsulfonyl fluoride, and 2 g/ml of (leupeptin, aprotinin, and pepstatin A) and were incubated with indicated antibodies overnight at 4 °C. After 24 h, lysates were coupled to protein A/G-agarose beads for 4 h at 4 °C. The beads were then collected by centrifugation at 3000 rpm for 3 min and washed three times using detergent-free radioimmunoprecipitation buffer. Phosphotyrosine of STIM1 was detected using a mixture of anti-PY20, anti-PY99 and anti-PY350 in equal proportion.

### Calcium imaging

An increase in intracellular Ca^2+^ was measured using the Ca^2+^- sensitive fluorescent dye Fura 2-AM as described. Briefly, HPAE cells were transfected with indicated constructs for 24 h or siRNA for 48 h respectively. Cells were then loaded with Fura 2-AM dye for 20 min, rinsed twice with Ca^2+^ free HBSS media and stimulated with thapsigargin 2 μM or thrombin 50 nM to determine Ca^2+^ release. Only YFP transfected cells were selected for Ca^2+^ measurement. Ca^2+^ entry was determined following addition of 2 mM Ca^2+^. In each experiment, at least 5 YFP-tagged cells were imaged for Ca^2+^ analysis.

### Preparation of Cells for Imaging and Image Analysis

Cells seeded on 35 mm glass bottom dishes were co-transfected with Orai1-mCherry and YFP-STIM1 or YFP-STIM1-Y361F mutant using FUGENE. After 24 h, cells were serum starved for an hour in 0.1% FBS after which cells were treated with 2 μM thapsigargin for 5 min or left untreated. Cells were fixed with 4% formaldehyde and imaged using a LSM 880 inverted confocal microscope (Carl Zeiss) equipped with a Plan-Apochromat 63x/1.4 NA oil immersion objective, an Argon (λ = 458, 488, 514 nm) and diode-pumped solid-state laser (λ = 561 nm), 2 photomultiplier tubes and Gallium arsenide phosphide detector. EYFP images were acquired with λ = 514-nm excitation and λ = 519–583 nm emission; mCherry images were collected with λ = 561 nm excitation and λ = 583–696 emission.

The 16-bit images were analyzed with Metamorph (Molecular Devices, Sunnyvale, CA) and ImageJ (NIH) software. Projected images were generated by collecting maximum pixel intensity of the in-focus frames into a single frame. The images were threshold to remove background fluorescence and used to create a mask image. Using the mask images, number of Orai1-mCherry and YFP-STIM1 clusters was scored with automatic “Analyze Particles” algorithm of ImageJ software and using cluster size of 3–100 pixels and circularity 0.1–1.0. The data were expressed as the number of clusters per area (μm^2^) of the cell.

Colocalization coefficient for Orai1-mCherry was determined using Z-stack images and Zen software (Carl Zeiss) according to the manufacturer’s instructions. Threshold images were used to set the vertical and horizontal crosshairs to separate clusters into quadrants. The colocalization coefficient was calculated as the sum of colocalized pixels divided by the total number of Orai1-mCherry clusters. All images were prepared for publication using Adobe Photoshop.

### Animals

All animals used in this study were approved by the Institutional Animal Care and Use Committee of (IACUC) University of Illinois. EC-STIM1^−/−^ mice were generated by crossing STIM1^fl/fl^ with Tie2-cre^58^,^59^. C57BL/6J was purchased from Jackson’s lab. All experiments were done on 6–8 weeks old mice.

### Liposomal Preparation and delivery of cDNA in the mouse lung

Liposomal delivery of cDNA into the mouse lung and lung vascular permeability measurements were performed in accordance with the IACUC. Cationic liposomes were made by mixing 9.5 ml of Chloroform and 315 μl of Dimethyl Dioctadecyl Ammonium Bromide (DDAB) with 200 μl cholesterol solution^43^,^60^. Lipid layer was formed by evaporating chloroform using a Rotavapor system at a speed of 105 rpm for 15–20 min at 37 °C. Lipid layer was scratched using 2 ml of 5% glucose and sonicated for 1 h at 42 °C. After filtering the liposomes through 0.45 micron filter 50 μM of cDNA was added drop wise to the 100 ul of liposome mixture and DNA-liposome complex was injected retroorbitally after anesthetizing the mice. After 48 h, mouse lungs were challenge with PAR1 peptide and lungs were harvested to determine lung vascular permeability and protein expression. All animals used in this study were approved by the Institutional Animal Care and Use Committee of (IACUC) University of Illinois.

### Lung vascular permeability

WT or EC-*STIM1*^*−/−*^ mice were anesthetized with an i.p. injection of ketamine (100 mg/kg) and xylazine (2.5 g/kg). PAR1 agonist peptide (TFLLRN-NH2) or control peptide (FTLLRN-NH2) (1 mg/kg body weight) were administered into mice through retroorbital route. After 30 min, left lungs were excised and completely dried in a 60 °C oven overnight for determining lung wet–dry ratio. All animals used in this study were approved by the Institutional Animal Care and Use Committee of (IACUC) University of Illinois.

### Generation of constructs

Generation of various constructs is described in Supplementary Information.

### Statistical analysis

Graphs were generated using GraphPad Prism (GraphPad Software, La Jolla, CA). Comparisons between experimental groups were made using one way ANOVA followed by *t*-test or Tukey test. Significance values are shown by *p < 0.05, **p < 0.01, and ***p < 0.001

## Additional Information

**How to cite this article:** Yazbeck, P. *et al*. STIM1 Phosphorylation at Y361 Recruits Orai1 to STIM1 Puncta and Induces Ca^2+^ Entry. *Sci. Rep.* **7**, 42758; doi: 10.1038/srep42758 (2017).

**Publisher's note:** Springer Nature remains neutral with regard to jurisdictional claims in published maps and institutional affiliations.

## Supplementary Material

Supplementary Information
