# Supplementary material for: STIM1 Phosphorylation at Y361 Recruits Orai1 to STIM1 Puncta and Induces Ca2+ Entry
Source: Sci Rep. 2017 Feb 20;7:42758. doi: 10.1038/srep42758 (PMC5316956; doi:10.1038/srep42758)
Supplement: Supplementary Information [file srep42758-s1.pdf]

Supplementary Information

STIM1 Phosphorylation at Y361 Recruits Orai1 to STIM1 Puncta and Induces Ca^{2+} Entry

Pascal Yazbeck[1], Mohammad Tauseef[1,2], Kevin Kruise[1], Md-Ruhul Amin[1], Rayees Sheikh [1],
Stefan Feske[3], Yulia Komarova[1#] and Dolly Mehta[1#]

Supplementary Information to

"STIM1 Phosphorylation at Y361 Recruits Orai1 to STIM1 Puncta and Induces Ca^{2+} Entry"

by Yazbeck et al

SUPPLIMENTARY EXPERIMENTAL PROCEDURES

Generation of Constructs

Human Orai1 cDNA was obtained from Addgene, after which it was subcloned into pAmCyan-N1 vector from Clontech via 5'-*XhoI* and *3'- BamHI*. Briefly, PCR primers were designed with flanking restriction sites, 5'-*XhoI* and *3'- BamHI,* to amplify the full length Orai1. The PCR fragment was then digested 5'-*XhoI* and *3'- BamHI* and ligated to the vector pAmCyan-N1 also digested at the same sites 5'-*XhoI* and *3'- BamHI.* The resulting plasmid is pAmCyan-N1-Orai1 with the Cyan tag at the C-terminus of Orai1. The subcloning of mCherry-Orai1 was done by digesting the pAmCyan tag out of the pAmCyan-N1-Orai1 plasmid, and insert into its place, the mCherry tag. To do so, PCR primers were designed to amplify mCherry from mCherry plasmid, pmCherry-C1, cat #632524, Clontech, with flanking restriction sites, *5'- AgeI* and *3'- NotI*. The PCR fragment was digested with*5'- AgeI* and *3'- NotI*. The vector pAmCyan-N1-Orai1 was digested also at the same sites *5'- AgeI* and *3'- NotI* to release the pAmCyan tag. The digested PCR fragment was then ligated to the digested vector. The resulting plasmid was mCherry-N1-Orai11. The PCR primers used were: for pAmCyan-N1-Orai1: Orai1-ATG-*XhoI*-F: 5'-AAAAAA*CTCGAG*GCCACCATGCATCCGGAGCCC-3' and Orai1-noStop-*BamHI*-R: 5'-GAAAAA*GGATCC*AAGGCATAGTGGCTGCCG-3'; and for mCherry-N1-Orai1: mCherry-*AgeI*-ATG-F: 5'-AAAAAA*ACCGGT*CGCCACCATGGTGAGCAAGGGC-3' and mCherry-STOP-*NotI*- R: 5'-GAAAAAGCGGCCGCTTTACTTGTACAGCTCGTCCAT-3'. Multi-step PCR was done to mutate the amino acids of wild type human STIM1 (GenBank Accession number NM_003156) in pEYFP-N1 vector (C-terminal tagged YFP vector from BD Biosciences- Clontech, Palo Alto, CA). The Y316F and Y361F mutation was done using a two-step PCR subcloning method, using pEYFP-N1-STIM1-WT (wild-type

STIM1) as the DNA template for PCR reactions. In the first PCR step, two sets of primers were used to generate PCR fragments “a” and “b” containing the YF mutated amino acid. In the second PCR step, the final PCR fragment “c” was generated by combining PCR fragments “a” and “b” in the PCR using a set of primers that included the flanking 5’-XhoI and 3’-Bam HI restriction sites. Primer sets used to generate fragment “a” were: 5’-GGAGTAACGGTTCTGGATAAAGGCAAACCAGCA-3’ hSTIM1-Y316F-F: 5’-GCGGAGCCGCCAAAAATTTGCTGAGGAGGAG-3’and hSTIM1-Y316F-R 5’-CTCCAACTCCTCCTCAGCAAATTTTTGGCGGCT-3’and hSTIM1-Y361F-F: 5’-GAGGTGGAGGTGCAATTTTACAACATCAAGAAGC-3’ and hSTIM1-Y361F-R 5’-TGCTTCTTGATGTTGTAAAATTGCACCTCCACC-3’ and hSTIM1-Y361D-F: 5’-GAGGTGGAGGTGCAAGATTACAACATCAAGAAGC-3’ and hSTIM1-Y361D-R: 5’-TGCTTCTTGATGTTGTAATCTTGCACCTCCACC-3’. Fragment ”c” was generated by using primers **hSTIM1-*XhoI*-F and hSTIM1-*BamHI*-R.** In the primers, the bold and italicized base pairs indicated restriction sites while the underlined base pairs indicated the mutated amino acid. The final PCR product “c” was then digested with XhoI and BamHI and ligated to pEYFP-N1 vector also digested with the same restriction enzymes to generate the resulting plasmid pEYFP-N1-STIM1mutants. The ligated reactions were transformed into DH5α Subcloning Effiency Competent Cells (ThermoFisher Scientific, Grand Island, NY). The transformed cells were plated onto LB plates supplemented with 25μg/ml Kanamycin (ThermoFisher). After overnight incubation at 37°C, single colonies were cultured in LB with 25μg/ml Kanamycin overnight. Plasmid DNA was obtained using GeneJet Plasmid Miniprep kit (ThermoFisher Scientific) and analyzed by restriction digestion to confirm the correct size of the vector (pEYFP-N1) and insert (STIM1). Further amplification of the plasmid DNA was done using NucleoBond Xtra Midi Kit (Clontech Laboratories, Mountain View, CA). The resulting plasmid was verified for DNA purity and identity by DNA quantification, gel analysis, and sequencing analysis.

Generation of VE-cadherin promoter (VECP) driven control vector and Y361F-STIM1 mutant was performed as described (Prandini et al., 2005). Briefly, YFP- (Yellow Fluorescent Protein) was PCR-

amplified using pEYFP-N1 as the DNA template (Clontech Laboratories, Mountain View, CA). YFP amplicon was digested and ligated to PGL4-VECP vector via *EcoRV*. The ligated reactions were transformed after which single colonies were screened and plasmid DNA was prepared as previously described. The YFP was engineered to be in frame and upstream of the luciferase reporter protein *luc2* in the PGL4.10 vector. The primers used for amplifying YFP were: YFP-ATG-*EcoRV*-F: 5'-TTTTTC*GATATC*AGCCACCATGGTGAGCAGCAAGGG-3' and YFP-NoSTOP-*EcoRV*-R: 5'-TTCTTC*GATATC*CTCTTGTACAGCTCGTCCATGCCG-3'; primers for amplifying YFP were YFP-start-*XhoI*-F: 5'-AAAAAA*CTCGAG*AGGCACCATGGTGAGCAAGG-3' was PCR-amplified using the pEGFP-N1 vector as the DNA template (Clontech Laboratories, Mountain View, CA). Primers used were STIM1-YFP-ATG-*EcoRV*-F: 5'-TTCTTC*GATATC*AGCCACCATGGATGTATGCGTCCG-3' and STIM1-YFP-NoStop-*EcoRV*-R: 5'-TTCTTC*GATATC*CTCTTGTACAGCTCGTCCATGCCG-3'.

Table S1. Secondary Antibody Used in This Study, Related to Figures 1A, 2A, 2B, 4A, 4B, 5A, 5B, S1A1, S1A2, S2A1 and S2A2.

Antibody	Species	Catalogue #	Application
HRP-conjugated anti-goat	Donkey	sc-2020	WB: (1:6000)<br>Figures 1A, 2B, 5B, S2B
HRP-conjugated anti-mouse	Donkey	sc-2318	WB: (1:10000)<br>Figures 1A, 2B, 4A, 5B, S1B
HRP-conjugated anti-rabbit	Goat	sc-2054	WB: (1:10000)<br>Figures 1A, 2A, 2B, 4A, 4B and 5B

WB, western blot
All secondary antibodies were from Santa Cruz Biotechnology.

Table S2. The siRNA Used in This Study, Related to Figures 2B, 2C, 4B, 4C.

Name	Target	Sense sequence	Catalogue#
Orai1 siRNA	Human	5'ACAUCGAGGCGGUGAGCAAT3'	AM16708
Pyk2 siRNA	Human	5'GAUGUGGUCCUGAAUCGUAtt3'	4390824

All siRNAs were from Ambion.

Table S3. Primary Antibodies Used in This Study, Related to Figures 1A, 2A, 2B, 4A, 4B, 5A, 5B, S1A, S1B, S2A and S2B.

Antibody	Species	Catalogue #	Application
PY20	Mouse	Santa Cruz (sc-508)	WB (1:1000)<br>Figures 1A, 2B, 5A and S1B
PY350	Rabbit	Santa Cruz (sc-18182)	WB (1:1000)<br>Figures 1A, 2B, 5A and S1B
PY99	Mouse	Santa Cruz (sc-7020)	WB (1:1000)<br>Figures 1A, 2B, 5A and S1B
Orai1	Rabbit	Santa Cruz (sc-68895)	WB (1:1000)<br>Figures 4A and 4B
Stim1	Goat	Santa Cruz (sc-79106)	WB (1:7000)<br>Figures 1A, 2B, 5A and S2B
Pyk2	Mouse	Cell Signaling (3480s)	WB (1:1000)<br>Figure 2A
p-Pyk2	Rabbit	Cell Signaling (3291s)	WB (1:1000)<br>Figures 2A and 5A
Actin	Goat	Santa Cruz (sc-1616)	WB (1:1000)<br>Figures 2A, 4B, 5A and S2B
GFP	Mouse	Santa Cruz (sc-9996)	WB (1:500)<br>Figures 4A, S1A, S1B and S2A

WB, western blot. The dilutions used for each application are shown.

SUPPLEMENTARY REFERENCES

PRANDINI, M. H., DREHER, I., BOUILLOT, S., BENKERRI, S., MOLL, T. & HUBER, P. 2005. The human VE-cadherin promoter is subjected to organ-specific regulation and is activated in tumour angiogenesis. *Oncogene*, 24, 2992-3001.

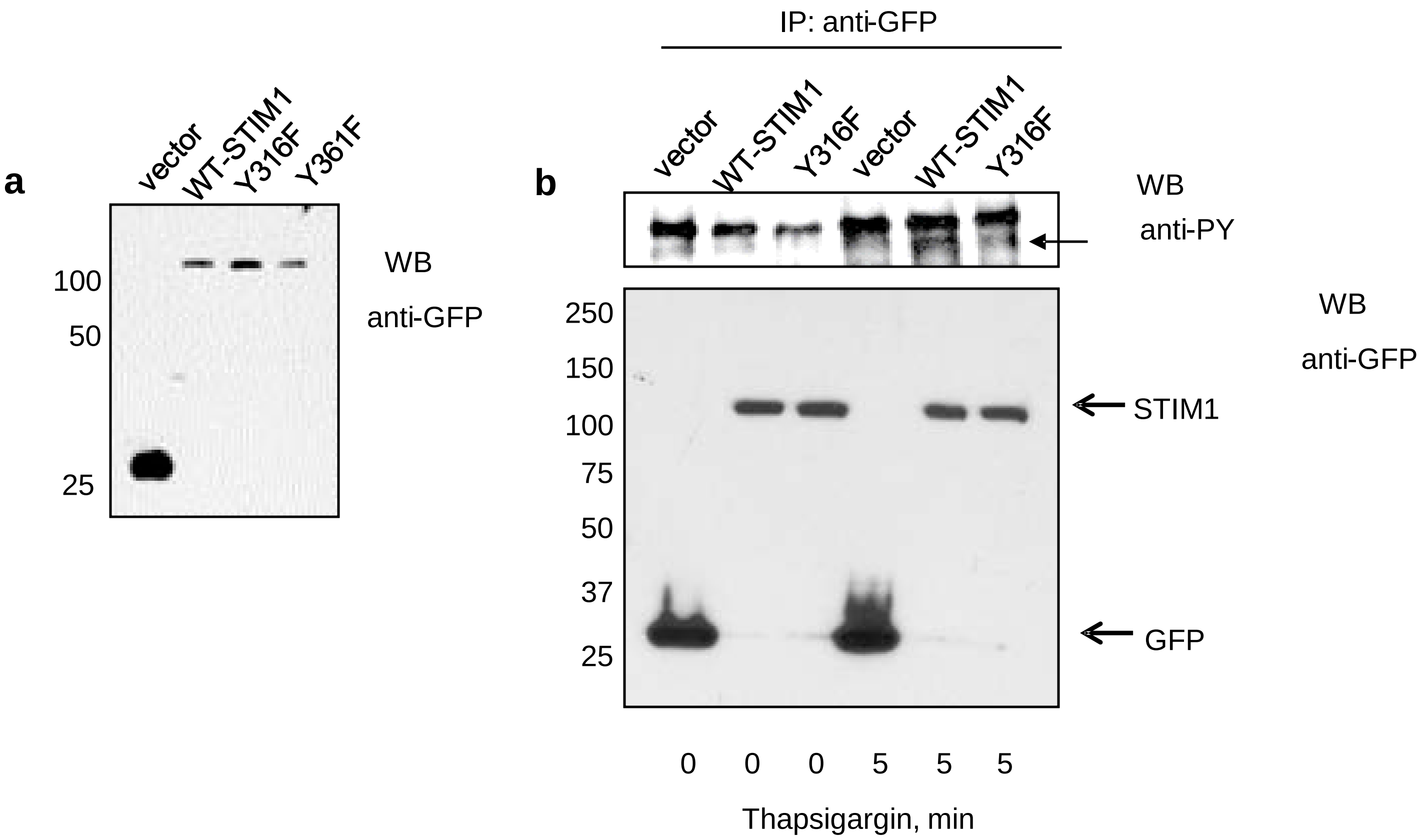

Figure S1. Phosphorylation of STIM1 at Y361 is required for triggering SOCE. Related to Figure 1. (**a)** HPAE cells were transfected with indicated cDNAs using FUGENE transfection reagent. After 24 h cells were lysed and equal amount of proteins were immunoblotted with anti-GFP antibody. Blot shows the expression of YFP, WT-STIM1,Y316F-STIM1 and Y361F-STIM1 mutants. (**b)** HEK cells were transfected with indicated cDNA for 48h after which cells were serum starved for 1-2 h in 0.1% FBS and stimulated with 2 μM thapsigargin for 5 minute or left unstimulated. Lysates were immunoprecipitated using anti-GFP antibody followed by immunoblotting for anti-tyrosine (anti-PY) antibody to assess phosphorylation of STIM1. Thapsigargin induced the phosphorylation of WT-STIM1 but not Y361F-STIM1. Anti-phosphotyrosine (anti-PY) antibody detects a non-specific band around 125 kd above phospho-STIM1.

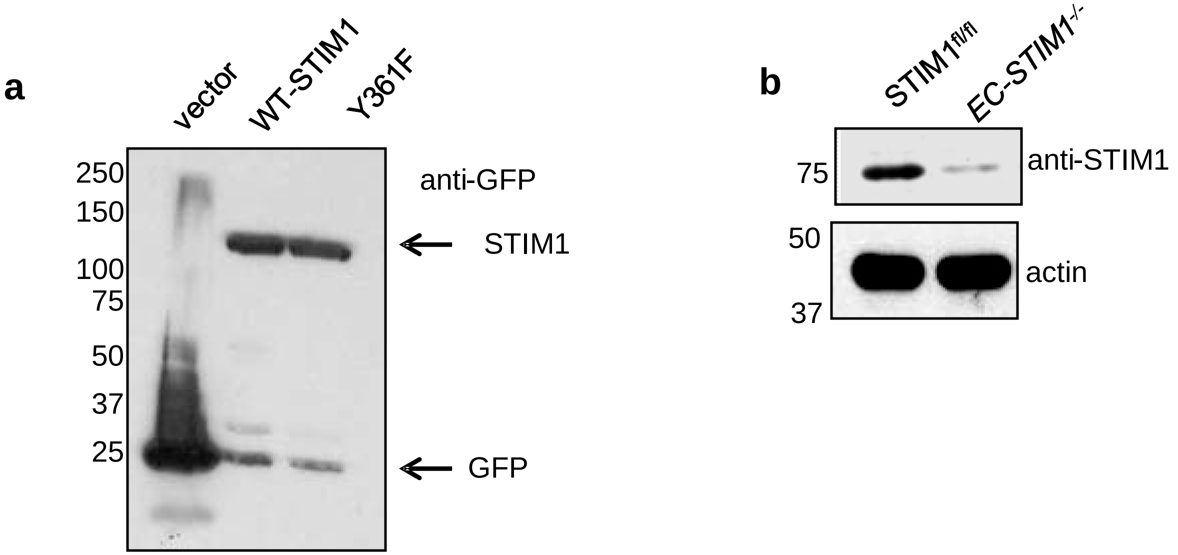

Figure S2. STIM1 phosphorylation at Y361 residue induces lung vascular permeability. Related to Figure 4. **(a)** Lungs from mice injected with liposome containing indicated cDNAs were harvested after 48h and equal amount of proteins were immunoblotted using anti-GFP antibody to detect the expression of WT-STIM1 or Y316F-STIM1 mutant. **(b)** Assessment of endothelial STIM1 expression. Lungs harvested from $STIM1^{fl/fl}$ or $EC\text{-}STIM1^{-/-}$ mice were homogenized and STIM1 protein expression was quantified using anti-STIM1 antibody. Actin was used as a loading control.

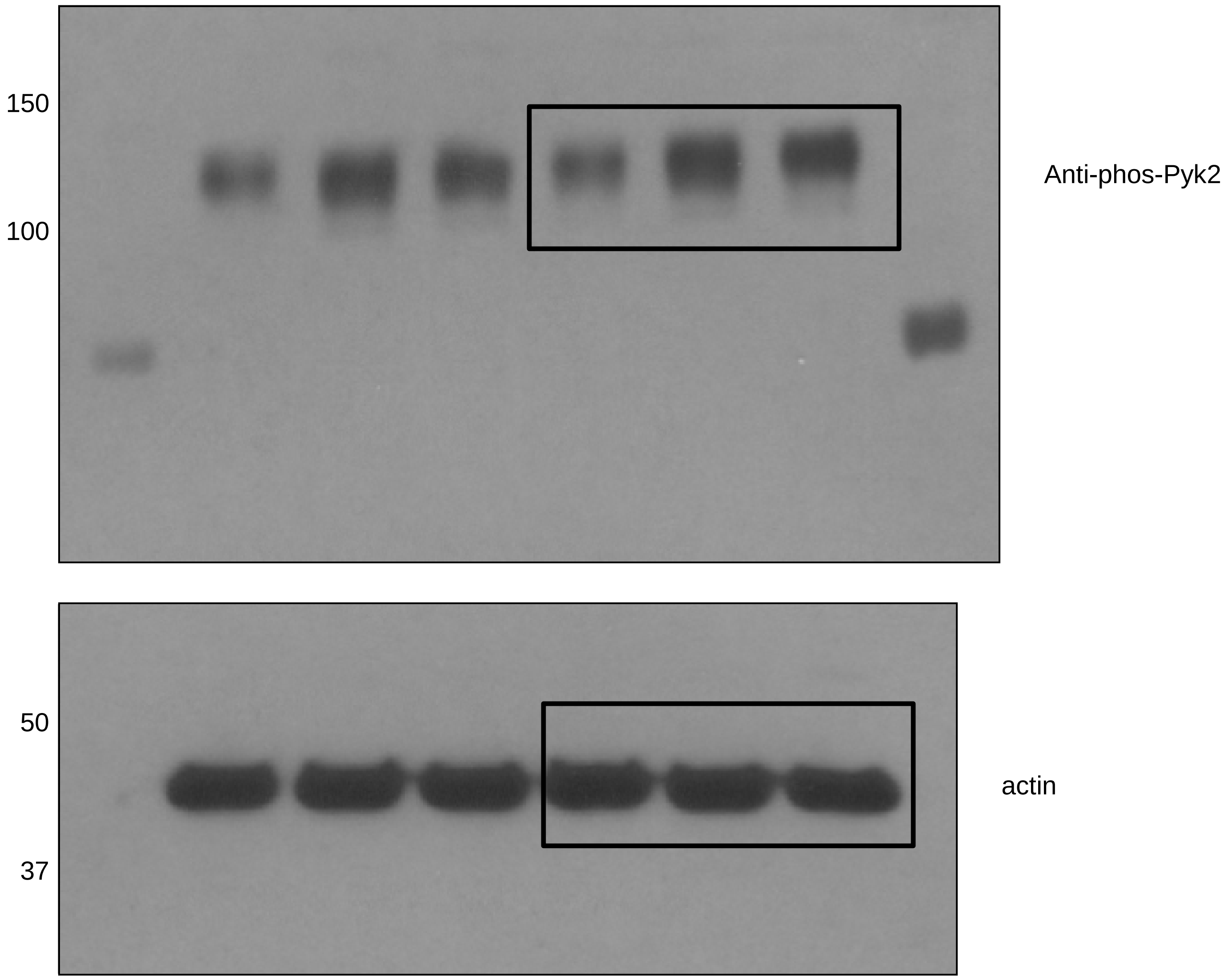

Figure S3: Full blots for Fig. 5A.
